# The watersports inclusion games - what are the benefits for volunteers?

**DOI:** 10.1192/j.eurpsy.2021.1235

**Published:** 2021-08-13

**Authors:** A. O’Flynn, J. Murphy, E. Barrett

**Affiliations:** 1 Department Of Child And Adolescent Psychiatry, School Of Medicine, University College Dublin, Dublin, Ireland; 2 Inclusion Games Officer, Irish Sailing, Dublin, Ireland; 3 Child And Adolescent Liaison Psychiatry, Children’s University Hospital, Dublin, Ireland

**Keywords:** inclusion, volunteerism, mental health, watersport

## Abstract

**Introduction:**

The Watersports Inclusion Games is an annual event organised by Irish Sailing and partners that provides an opportunity for individuals of all abilities across the physical, sensory, intellectual and learning spectrums and those experiencing barriers accessing mainstream sport to partake in a range of watersports. 79 volunteers from the 2019 cohort responded to a pilot survey to assess the benefits for volunteers at the event.

**Objectives:**

This project aims to assess this data in the context of current knowledge about the benefits for volunteers in inclusive sport.

**Methods:**

Literature review used the PEO keyword framework in medical and psychological databases, as well as grey literature. Data was collected using SurveyMonkey, quantitative data was analysed using Survey Monkey and SPSS, and qualitative themes were analysed using SurveyMonkey and Excel.

**Results:**

Only one article exploring the benefits for volunteers in inclusive watersports was identified during literature review. This pilot survey analysis is the first on this topic in Ireland, and the largest sample of volunteers in inclusive watersport that we are aware of internationally. Thematic analysis finds that volunteers at this event are primarily motivated by altruistic motives, while the benefits they perceive include both personal enjoyment and growth, and seeing the enjoyment of other participants.

**Conclusions:**

This project demonstrates that inclusive watersports can have many benefits for volunteers. The findings of this study can contribute to the evidence base on the benefits of inclusive sport for all those involved, while also identifying an opportunity for further study on volunteerism in inclusive sport, particularly adaptive watersports.

**Conflict of interest:**

Ms O’Flynn reports a scholarship from the Health Research Board for this project, Dr Barrett has nothing to disclose, Ms Murphy reports to be the Inclusion Games Office, and thus responsible for the organisation of the Watersports Inclusion Games.

